# Composite Survival Index to Compare Virulence Changes in Azole-Resistant *Aspergillus fumigatus* Clinical Isolates

**DOI:** 10.1371/journal.pone.0072280

**Published:** 2013-08-26

**Authors:** Eleftheria Mavridou, Joseph Meletiadis, Pavol Jancura, Saiden Abbas, Maiken C. Arendrup, Willem J. G. Melchers, Tom Heskes, Johan W. Mouton, Paul E. Verweij

**Affiliations:** 1 Department of Medical Microbiology, Nijmegen Institute for Infection, Inflammation and Immunity, Nijmegen, The Netherlands; 2 Nijmegen Institute for Infection, Inflammation and Immunity, Nijmegen, The Netherlands; 3 Laboratory for Clinical Microbiology, Attikon University General Hospital, Athens, Greece; 4 Department of Microbiological Surveillance and Research, Statens Serum Institut, Copenhagen, Denmark; 5 Institute for Computing and Information Sciences, Faculty of Science, Radboud University, Nijmegen, The Netherlands; Geisel School of Medicine at Dartmouth, United States of America

## Abstract

Understanding resistance to antifungal agents in *Aspergillus fumigatus* is of increasing importance for the treatment of invasive infections in immunocompromised patients. Although a number of molecular resistance mechanisms are described in detail, the potential accompanying virulence changes and impact on clinical outcome have had little attention. We developed a new measure of survival, the composite survival index (*CSI*) to use as a measure of the virulence properties of *A. fumigatus*. Using a novel mathematical model we found a strong correlation between the *in vitro* growth characteristics and virulence *in vivo* expressed as *CSI*. Our model elucidates how three critical parameters (the lag phase (*τ*), decay constant (*λ*), and growth rate (*ν*)) interact with each other resulting in a *CSI* that correlated with virulence. Hence, strains with a long lag phase and high decay constant were less virulent in a murine model of invasive aspergillosis, whereas high virulence for isolates with a high *CSI* was associated *in vitro* with rapid growth and short lag phases. Resistant isolates with *cyp51A* mutations, which account for the majority of azole resistant aspergillosis cases, did not show a lower virulence compared to azole-susceptible isolates. In contrast, the *CSI* index revealed that a non-c*yp51A*-mediated resistance mechanism was associated with a dramatic decrease in *CSI*. Because of its predictive value, the mathematical model developed may serve to explore strain characteristics in vitro to predict virulence *in vivo* and significantly reduce the number of experimental animals required in such studies. The proposed measure of survival, the *CSI* can be used more in a general form in survival studies to explore optimal treatment options.

## Introduction

*Aspergillus fumigatus* is responsible for the majority of invasive fungal infections in immunocompromised patients. Timely treatment with antifungal drugs is essential for the management of this disease and numerous efficacy studies have been carried out both in animals and humans to support evidence-based treatment choices [1], [2], [3]. To date, most investigations into antifungal efficacy have concentrated upon growth inhibition (MIC) whereas the role of fungal virulence has been largely ignored. The role of virulence as a factor in the disease outcome of patients with aspergillosis has hardly been considered, despite data obtained for other microorganisms indicating that the factor virulence may affect treatment [4], [5], [6].

The recent emergence of acquired resistance of *A. fumigatus* to medical triazoles [7], [8], [9], [10], [11], [12], [13], [14], has drawn attention to the question whether the evolution of azole resistance has any impact on the ability of the fungus to cause infection in man, and subsequently, on the clinical outcome. A link between azole drug resistance and the virulence of *A. fumigatus* was first demonstrated by Willger and colleagues [15]. Loss of SrbA, a sterol regulatory element binding protein resulted in growth incapacity of *A. fumigatus* and inability to cause fatal infections in two murine models of invasive pulmonary aspergillosis [16]. Further examination of the SrbA null mutant revealed that SrbA played a critical role in resistance to the azoles. Moreover, we recently reported reduced virulence in clinical *A. fumigatus* isolates that had become resistant to azoles during azole therapy [17]. A reduction of virulence in the above-mentioned studies was observed in non-*cyp51A* gene associated resistance mechanisms, while *cyp51A*-associated short nucleotide polymorphisms (SNPs) are the most prevalent resistance mechanism in clinical *A. fumigatus* isolates. Numerous SNPs in the *cyp51A* gene have been reported in clinical *A. fumigatus* isolates [18], [19], [20], [21], which confer increased minimal inhibitory concentrations (MIC) for azoles *in vitro* and reduced azole efficacy *in vivo* [22], [23]. Although *A. fumigatus* isolates with *cyp51A* mutations cause invasive aspergillosis in humans, indicating their ability to cause infection, quantitative estimates of *in vivo* virulence of isolates harboring those mutations are lacking [24].

A standardized animal model to compare the virulence of different *A. fumigatus* isolates is absent. It has been shown previously that variation in virulence between *A. fumigatus* isolates exists in a murine infection model but this study did not report susceptibility data [25] and other models previously used for measuring virulence reported unsatisfactory results [26].

In the present study, we investigated whether *cyp51A*-associated azole resistance mechanisms favored a gain or loss of virulence in *A. fumigatus.* To that purpose, we used a simple *in vivo* non-neutropenic murine model of disseminated aspergillosis. In particular, we explored the effects of *in vitro* growth characteristics on survival and developed a novel mathematical model. We propose and describe a new composite survival index (*CSI*) that enabled the prediction of survival in the animal model. The *CSI* was subsequently used to determine the impact of resistance mechanisms on the virulence of *A. fumigatus*.

## Materials and Methods

### Isolates

Thirty clinical *A. fumigatus* isolates from different patients and hospitals were used in this study (Table S1). Microsatellite genotyping showed no genetic relationship among all isolates (Table S1). Ten isolates were defined as wild type based on the *in vitro* susceptibility profile and absence of mutations in the *cyp51A* gene (Table S1). Twenty isolates were defined as non-wild type based on the *in vitro* susceptibility profile and the presence of mutations in the *cyp51A* shown to be associated with azole resistance.

From the collection of the aforementioned 30 isolates, we used in total 15 clinical isolates for the *in vivo* studies; three WTs (V28–29, V52–76, AZN 8196), three isolates were used that harbored the TR_34_/L98H resistance mechanism, which are believed to be selected through exposure to azole fungicides in the environment [9]. This mechanism was found to be the dominant resistance mechanism in clinical isolates in the Netherlands, other European countries and in Asia [27], [28], [29] and has also been found in azole-resistant isolates recovered from the environment [8], [30].

Five isolates harbored SNPs in *cyp51A*, which were selected during azole therapy, including substitutions at codon M220 (28–77 with M220I, v13-09 with M220V, v59-07 with M220K), codon G54 (G54W, isolate v59–73), and codon 138 (G138C, isolate v59–72). In addition, four isogenic *A. fumigatus* isolates used were cultured serially from a single patient (isolates S1, S2, R1, and R2) [17]. The patient had chronic granulomatous disease (CGD) and was treated with multiple regimens of antifungal azoles for a chronic pulmonary *Aspergillus* infection. On antifungal therapy, the azole susceptibility changed from a wild-type phenotype (S1 and S2) to a resistant phenotype (R1 and R2). Although the expression of the *cyp51A* gene in the isolates R1 and R2 was elevated compared with the S1 and S2 isolates, no SNPs were found in *cyp51A* [17]. All isolates were stored in 10% glycerol broth at −80°C and were revived by subculturing on Sabouraud dextrose agar (SAD) supplemented with 0.02% chloramphenicol for 5 to 7 days at 35°C.

### Molecular Analysis

The morphological identification of the *A. fumigatus* isolates was confirmed by sequencing of the β-tubulin and calmodulin genes, as described previously [8]. Genetic relationships of all isolates were determined by microsatellite genotyping (Table S1) [31]. This previously described assay relied on the variability of STRs in the *A. fumigatus* genome. Three trinucleotide and three tetranucleotide repeats of six different loci were amplified by using fluorescently labeled primers. The amplified DNA fragments were determined by the addition of the GeneScan LIZ [500] marker and were analyzed with the Applied Biosystems 3730 DNA software system. The assignment of repeat numbers in each marker was determined from GeneScan data by using Peak Scanner version 1.0 software (Applied Biosystems). The cyp51A coding region and its promoter were sequenced as previously described [18], [32].

### *In vitro* Studies

Antifungal susceptibility testing was performed based on the M38-A2 method of the Clinical Laboratory Standards Institute (CLSI) by using a microbroth dilution format and MICs of voriconazole, posaconazole, and itraconazole were determined as the lowest drug concentration resulting in no visible growth after incubation for 48 h [33]. *In vitro* growth curves of the thirty isolates were determined by using a previously described microbroth kinetic system [34], [35]. OD measurement can be used to quantify *Aspergillus* growth and it describes changes in fungal biomass. Briefly, 96-well microtitration plates were inoculated with 200 µL of RPMI1640 with 0.165 M MOPS containing 2.5×10^4^ conidia. To prevent evaporation, the vertical margins of the microtiter plates were sealed with autoclave sterilization tape. After agitation for 15 s the suspensions were incubated at 37°C inside a plate reader (Rosys Anthos ht3; Anthos Labtec Instruments GmbH, Salzburg, Austria) for 90 h. The optical density at 405 nm (OD) was automatically recorded for each well every 15 min. The changes in OD over time were used to generate growth curves for each isolate in triplicate (OD-growth). All studies were conducted twice. The mean value was used for statistical analyses.

### *In vivo* Studies

To compare the *in vivo* virulence of each *A. fumigatus* isolate, a previously described experimental murine model of disseminated aspergillosis was used [17], [22]. A subset of 15 out of the 30 *A. fumigatus* strains was chosen based on the azole resistance mechanism and the duration of the lag phase, which was recently implicated as an important marker to predict virulence [17]. A total of 660 outbred CD-1 female mice (20–25 g, 4–5 weeks old) were randomized into 60 groups (n = 11 per group). Each of these groups was infected intravenously by the tail vein with four different inocula of the aforementioned isolates: concentrations of 1×10^6^, 5×10^6^, 1×10^7^, and 5×10^7^ CFU per mouse. Post-infection viability counts of the injected inocula were determined to ensure that the correct inoculum had been injected. Mortality was monitored for 15 days. On day 15 post-infection, all remaining mice were humanely euthanized by cervical dislocation. To assess the variability of *in vivo* studies, *A. fumigatus* isolates with different azole resistance mechanisms (the wild type, G54W, TR_34_/L98H__v52–35_, and the M220I isolate) were tested in triplicate at a low (1×10^6^ CFU per mouse) and a high (5×10^7^ CFU per mouse) inoculum. The differences in percentage of survival and MST were <10% and <1 day among replicates, indicating very high reproducibility.

The animals were housed under standard conditions with water and food supplied ad libitum. Animal studies were carried out in strict accordance with the recommendations of the European Community (Directive 86/609/EEC, 24 November 1986). The protocol was approved by Animal Welfare Committee of the Radboud University (RU-DEC 2007-106). All efforts were made to minimize suffering. To prevent severe discomfort and substantial distress, throughout the whole study mice were monitored and clinically inspected several times day and night. The experiment was immediately terminated if the humane endpoints were not fulfilled. Symptoms of discomfort and distress included loss of weight (15% loss within 72 h or 20% within 48 h); very high or very low body temperature; desiccation; lethargy; hyperactivity in terms of spinning around in circles constantly; eyes turning black; reduced mobility, and therefore, mice are unable to reach food or drink; or generally reduced food/water intake. All stressed animals were sacrificed by cervical dislocation. The murine model of disseminated aspergillosis is well established in our laboratory. Therefore, the veterinary experts and investigators are able to recognize the clinical symptoms in the animals at a very early stage and can ensure early termination before the humane endpoints are reached without affecting interpretation of the experimental results.

### Modeling of the *in vitro* Growth in *A. fumigatus*

*In vitro* growth curves were analyzed with nonlinear regression analysis based on a novel mathematical model of fungal growth. Previous reports suggested Malthusian fitness as a possible measure model of fungal fitness [36]. This function assumes that the size of the population is limited by an asymptotic maximum (plateau). However, because the previous and our present growth kinetic experiments showed that a plateau phase was not reached, we set out to define a new model that better described the observed growth [34], [35], [37]. This model incorporated a piecewise function of 1) the lag phase (*τ*), which corresponded to no OD-growth changes observed until the hyphal length reached 70 µm; and 2) the growth phase of the fungus, which corresponded to OD-growth changes post *τ*. The lag phase was described as a constant function. The growth phase was modeled by the function composed of two main factors: linear OD growth and exponential decay. With these factors, the model reasonably captures the dynamics of the growth phase and the death phase which are constantly and simultaneously present during the life cycle of a cell population which is composed of a heterogeneous population of aging cells. The underlying assumptions of our model are currently utilized in prediction models for other cell types and organisms which take into account the variability in the aging process of the cells. To the best of our knowledge there has been no similar model for filamentous fungi allowing the modeling of the whole life cycle.

Thus, the proposed model for simulating the growth of *A.* *fumigatus in vitro* is defined by Equation 1:
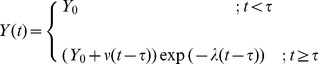
(1)where *Y(t)* represents the fungal biomass assessed based on the optical density at time *t*. For the mathematical growth function parameters, *Y_0_* corresponded to the OD value for the period of the lag phase, time τ is the lag phase duration in hours, ν is the growth rate expressed in OD×h^−1^, and λ is the growth decay constant expressed in h^−1^. The use of linear components in a nonlinear equation is a common approach which increases the flexibility of the equation in order to capture complex phenomena like Aspergillus dynamic growth. In general, this growth model assumes linear growth of the population (linear component), whereas, at the same time, cell death would occur due to ageing and nutrient consumption (exponential component). The proportion of cell death would increase over time.

### Clustering Analysis of Strains Based on the Fungal Growth

To identify whether there were differences in fungal growth between the strains, clustering analysis was performed on their growth parameters (*τ,ν,λ*) as follows: Each parameter was standardized by computing its *z-*score. Then, since the number of clusters was unknown, we created an agglomerative hierarchical cluster tree by applying the centroid linkage method (UPGMC) with the Euclidean distance.

The quality of agglomerative clustering was assessed by the cophenetic correlation coefficient (CPCC). The significance of clustering was estimated by computing the probability of obtaining the CPCC by chance as follows: Given a group of *N* strains, we generated 10^6^ random samples of *N* objects (“random” strains) with coordinates from the same range as the given *N* strains. Then, parameters of each random sample were *z*-score standardized and the objects were clustered by the same agglomerative technique as the original data. CPCC was computed for each random sample (CPCC_i_). As a result, we accumulated the distribution of CPCC_i_s for these clusterings (C^R^ = {CPPC_i_ | i ∈ {1,2,…,10^6^} }) and we counted how many times CPCC_i_ was equal to or greater than the cophenetic correlation CPCC_S_ of the clustering original strains (C = {CPCC_i_ ∈ C^R^ | CPCC_i_ ≥ CPCC_S_ }). Then, the empirical *p* value of the original clustering was determined as
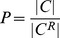
If clustering of the original strains was significantly better than clustering of random samples (*P*<0.05), the derived clustering structure had a low probability of being obtained by chance.

### The *CSI*: a New Composite of Survival

For *in vivo* studies, survival data for each inoculum is analyzed based on the Kaplan Meier method (log-rank test), and *SUR%* and *MST* is determined. *SUR%* is calculated as the number of survivors over the total number of infected animals at the end of the experiment. *MST* is the time at which the fractional survival equals 50% (GraphPad Prism, version 5.0). For inocula where 50% mortality is not observed within the predefined time interval, *MST* is considered as maximum *MST* (*MST_max_*) equal to the day of termination of the experiment.

Although, *SUR%* is considered to be a measure for estimating virulence in general, it does not take into account *MST*, which is also an outcome of Kaplan Meier analysis. We therefore propose a new composite index of these two parameters of survival, the *CSI*. *CSI* is defined to include both terms as follows:
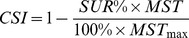
(2)where *MST_max_* is the length of the *in-vitro* experiment, which equals to the day of termination of the experiment. The denominator of the second term is a normalizing constant that restricts *CSI* between 0 and 1. *CSI* corresponds to the degree and rate of mortality, for which high mortality corresponds to high *CSI*. The *CSI* itself is used here as a direct measure of virulence, but can be applied as an index value for any survival curve.

### Correlation between Fungal Growth and *CSI*

A multiple logistic regression analysis was employed to determine the relationship between virulence markers and fungal growth-curve parameters. Logistic functions were used to fit three different virulence markers as follows
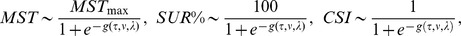
in which 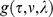 is the following function of growth parameters:
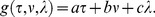
(3)
*a, b*, and *c* are regression coefficients. Virulence was quantified by using *MST*, *SUR%*, and *CSI*. Thus, for all virulence measurements (response-dependent variables), the regression coefficients were estimated independently. In addition, the regression for each response model was cross-validated by using Stein formula [38] of adjusted 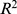 to assess the loss of predictive power of each response model.

### Comparison of the Virulence-marker Models

The two virulence markers with the best goodness of fit were compared to assess which was explained better by the growth parameters. To this end, we computed standardized residuals for each of the two response models using leave-one-out cross validation.

Given a model, one response value was omitted and the multiple logistic regression analysis was performed on the remaining response values to estimate the regression coefficients. Afterwards the removed response was predicted by the logistic function by using the estimated coefficients. The standardized residual (*R*) of the predicted value (*V_pred_*) and the observed value (*V_obs_*) of the omitted response was computed by using the formula
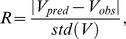
in which 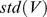 was the sample standard deviation of all response values. The procedure was repeated for each response value. For each strain, we compared the standardized residuals of response values of both models: the lower the standardized residuals, the better the fit.

### Construction of a General Model

The logistic function-based model given by Equation 4 was used to incorporate the four-dimension dataset as a general model in which *CSI* was associated with growth-curve parameters (*τ,ν,λ*) and inoculum size, *Φ*:
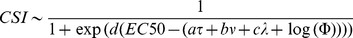
(4)in which EC50 is a parameter associated with a *CSI* value of 0.5.

## Results

### Phenotype and *in vitro* Growth Characteristics

**Figure 1:**
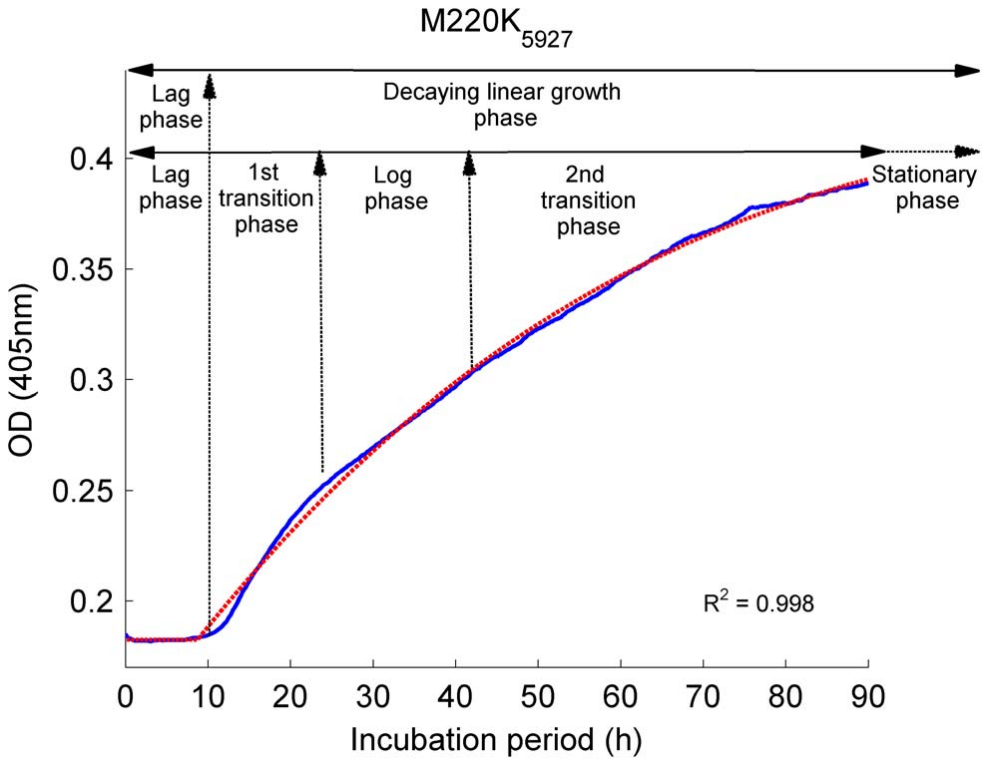
Modeling of the *in vitro* growth in *A.* *fumigatus*. In the figure is depicted an example of model fit to a single isolate. The growth is characterized by five phases: the lag phase, where no OD-growth changes were observed; the 1st transition period, where the OD change rate increased; the log phase, where the OD change rate was maximal; and the 2nd transition period, where the OD change rate decreased to reach a stationary phase. The output of the mathematical model developed herein shows that two phases are critical to measure fungal growth *in vitro*: a) the lag phase and b) the linear OD decaying growth phase, which includes the 1st and 2nd transition periods and the log phase. The proposed model (Equation 1) for simulating the growth of *A. fumigatus in vitro* (blue line) fitted well to the observed growth curves (red line).

The *in vitro* susceptibility phenotype to itraconazole, posaconazole, and voriconazole, the underlying resistance mechanism, and the short tandem repeat (STR) profile of the 30 clinical *A. fumigatus* isolates are shown in Table S1. The recently proposed interpretative breakpoints were used to categorize the phenotypes of the isolates [39]. For each isolate, the growth curve was characterized using a microbroth kinetic system [34], [37]. In this system, microscopic examination has confirmed that an increase in the optical density corresponds to the multicellular development of *A. fumigatus* [37]. Previously multiple phases (lag phase, log phase, and two transition phases) were identified to describe the growth of *A. fumigatus in vitro* [37], however our novel mathematical model identified only two phases; the lag phase and the decaying linear OD growth phase, the latter including the log and two transition phases (Fig. 1, Equation 1). The proposed growth function describes the first phase through the duration of the lag phase (*τ*), and the second phase by the linear OD growth rate (ν) and the decay constant (*λ*). The stationary phase was not observed during the incubation period of 90 h. The mathematical model of growth fitted well to the observed growth curves (Fig. S1) with *R* ^2^ values ranging from 0.98 to 0.99 with no zero value within the 95%CI of the model parameters for all isolates (Table S2). The correlation study of the growth parameters (*τ,ν,λ*) revealed that two parameters, *ν* and λ, exhibit a high and statistically significant linear correlation (Table 1). Despite this correlation, the *CSI* measure using both growth parameters can be still applied as a predictive tool which is verified by F-test on nested models of *CSI* (Table 4).

**Table 1:** Sample correlation coefficients between the growth parameters (*τ,ν,λ*).

CorrelationCoefficients	*τ*	*ν*	*λ*		p-values	*τ*	*ν*	*λ*
***τ***	1	−0,0022	0,1534		***τ***	1	0,9938	0,5851
***ν***	−0,0022	1	0,8651		***ν***	0,9938	1	0,000031
***λ***	0,1534	0,8651	1		***λ***	0,5851	0,000031	1

**Table 4:** The F-test statistics of nested CSI models compared to the original *CSI* model.

Growth parameters kept	F	p-value
***τν***	27.76	0.0002
***τλ***	134.14	7.17×10^−08^
***νλ***	43.36	2.59×10^−05^
***τ***	78.33	1.30×10^−07^
***ν***	54.11	9.89×10^−07^
***λ***	67.87	2.87×10^−07^
***τνλY_0_***	4.61	0.055

The last row demonstrate F statistics when the original model is nested with respect to the extended model by adding Y_0_ into the linear term g(*τ,ν,λ*).

### Differential Survival Outcomes Reflect a Strain-dependent Virulence Distinction

To explore whether there was a variation in virulence of our strains *in vivo,* we performed survival experiments with 15 *A. fumigatus* strains using 4 different inocula for each strain. Table 2 depicts differential survival rates for each strain of mice groups inoculated with the same lethal or sublethal inoculum. Susceptibility to infection was most prominent in animals infected with an inoculum of 5×10^7^ CFU and no clear differences in percentage of survival (*SUR%*) and median survival time (*MST*) were observed, with exception of two strains. In mice infected with azole-resistant isolates R1 and R2, 100% mortality occurred in a time period that was significantly longer than the other groups (*p*<0.05). Lower mortality and longer MST was also observed at the inoculum of 10^7^ CFU (*p*<0.05). In contrast, mice infected with the parental S2 rapidly succumbed at a dose of 10^6^ conidia and 5×10^6^, while these concentrations showed a sublethal outcome in the case of infection with wild type or any of the other mutants. Moreover, both highest inocula concentrations resulted in lowest survival in shortest time compared to all other groups (Table 2). Similarly, the second lowest *SUR%* with short *MST* was found for the groups infected with the G54W mutant, however, mainly for the two lowest inocula.

**Table 2:** Differential survival outcome reflects strain-dependent virulence distinction.

Isolates	Inoculum 10^6^ CFU		Inoculum 5×10^6^ CFU		Inoculum 10^7^ CFU		Inoculum 5×10^7^ CFU		
	*SUR%*	*MST*	*SUR%*	*MST*	*SUR%*	*MST*	*SUR%*	*MST*	
WT _(AZN 8196)_	100	15	72.72	15	18.18	8	0		3
WT _(V52–76)_	63.63	15	36.36	12	9.09	4	0		2
WT _(V28–29)_	100	15	63.63	15	0	6	0		2
TR_34_/L98H _(V52–35)_	90.90	15	45.45	8	18.18	8	0		3
TR_34_/L98H _(V45-07)_	100	15	27.27	11	45.45	11	0		2
TR_34_/L98H _(V61–76)_	100	15	18.18	8	0	4	0		2
M220I__V28–77_	81.81	15	63.63	15	0	7	18.18		5
M220K__V59-27_	100	15	63.63	15	45.45	12	0		2
M220V__V13-09_	81.81	15	9.90	10	9.09	9	0		2
G54W__V59–73)_	45.455	8	0	8	0	4	0		3
G138C__V59–72_	90.09	15	54.54	15	0	6	0		2
S1__V67-38_	90.09	15	63.63	15	36.36	15	0		2
S2__V67-37_	36.36	5	0	2	0	2	0		1
R1__V67-36_	100	15	90.09	15	90.09	15	0		6
R2__V67-35_	100	15	100	15	63.63	15	0		6

As our survival data suggested a wide variation in the virulent traits of some strains-, we next addressed the question if the observed variation in *SUR%* and *MST* could be correlated with characteristics of the *in vitro* growth curves. Furthermore, to improve the sensitivity to detect *in* *vivo* virulence differences, we developed a dynamic marker, the Composite Survival Index (*CSI*), which describes the rate (*MST*) and extent of killing (*SUR%*) (Equation 2).

### Cluster Analysis Reveals Heterogeneous Growth within the Wild Type and Azole-resistant Populations

**Figure 2:**
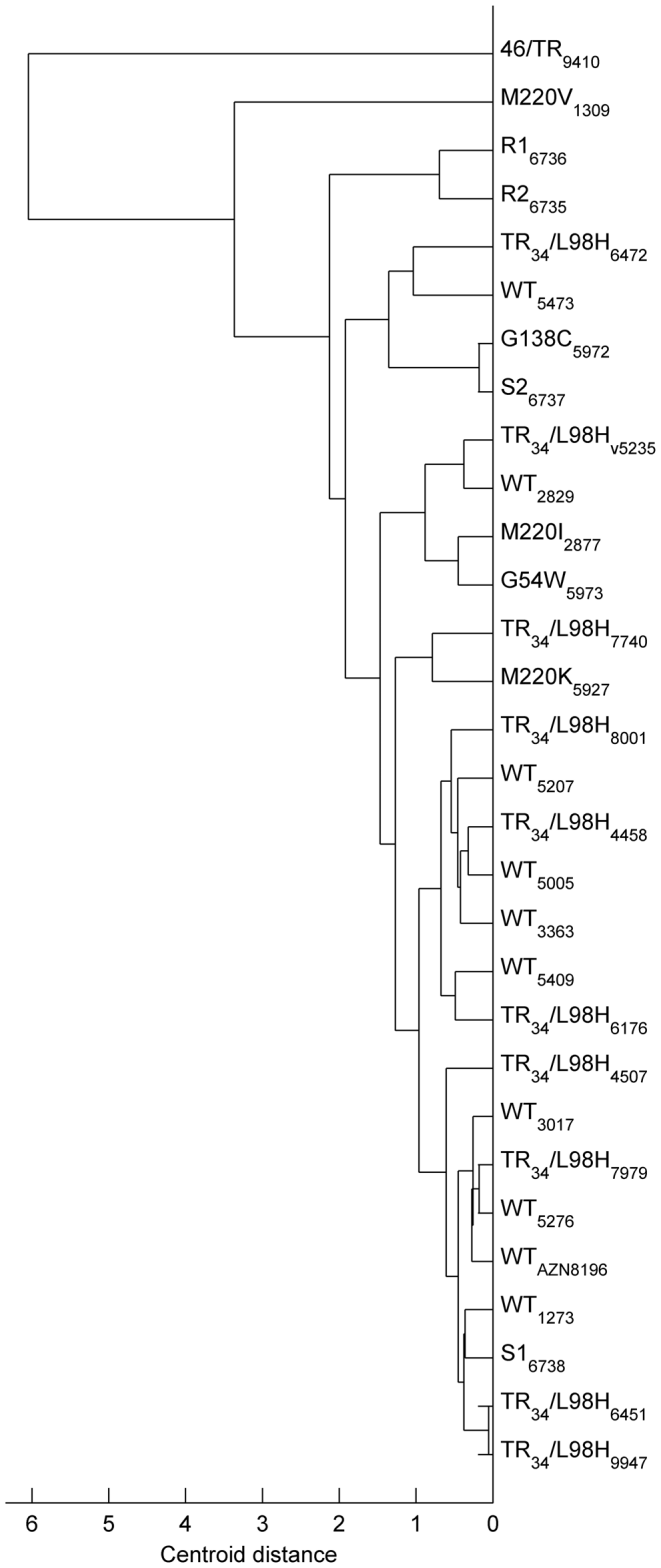
Clustering analysis of strains based on the fungal growth. The growth-curve parameter (*τ,ν,λ*) estimates of 30 strains revealed interstrain variability in growth within the WT groups and the non-wild-type isolates. The subscripts indicate the ID numbers of each *A. fumigatus* strain used for the current study.

A clustering approach was applied to the growth-curve parameter (*τ,ν,λ*) estimates of all strains to explore primary differences in growth between the wild type and the non-wild type isolates more accurately (Fig. S2 & Fig. 2). Statistical analysis revealed that clustering was significantly better than that found for a random population (CPCC = 0.922, *p*<10^−6^). Interestingly, there was no single-cluster formation of the wild type population, indicating that the wild type strains did not share similar growth characteristics. Similarly, no single cluster was observed for any of the strains harboring *cyp51A* mutations (Fig. 2).

### Virulence in *A. fumigatus* is Related to Growth

**Figure 3:**
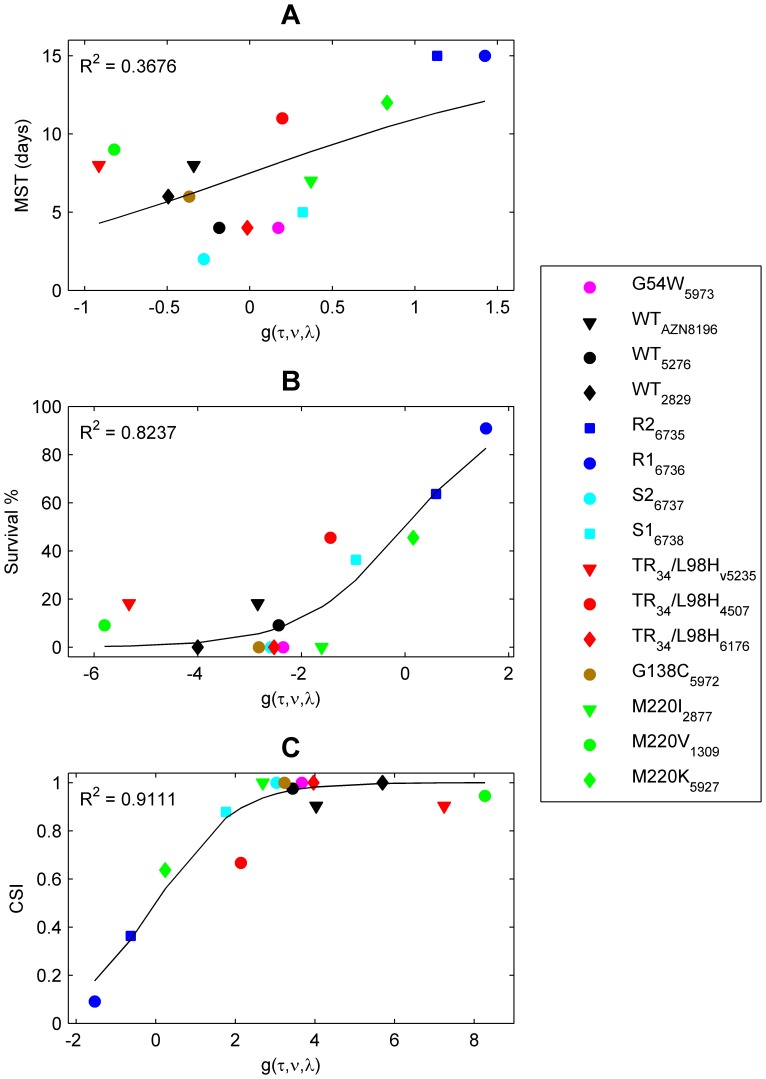
Relationship between the virulence markers *MST*, and *SUR %* and *CSI* and the function *g(τ,ν,λ)*. The logistic function was used to fit three different virulence markers: the median survival time (*MST*, panel A), the survival percentage (*SUR %*, panel B) and the composite survival index (*CSI*, panel C). No *in vitro*-*in vivo* correlation was found between *MST* and *g(τ,ν,λ)*, whereas a strong correlation was found for the other two virulence markers. The symbols correspond to the observed *CSI* values, while the solid line is the outcome of the prediction model.

To determine whether growth of *A. fumigatus* was associated with virulence, we investigated the relationship between *MST*, *SUR%*, the new proposed Composite Survival Index (*CSI*) and the growth properties (*τ,ν,λ*) of each strain. This index incorporates both *SUR%* and *MST* and may be viewed as an indicator of virulence of a strain (Equation 2). Multiple logistic regression analysis was used to fit *in vitro* growth characteristics *τ*, *ν*, and λ to all three virulence markers (Fig. 3). For each virulence marker, the fitting procedures estimated different regression coefficients (*a*, *b*, *c*) (Table 3).

**Table 3:** Descriptive statistics of fitting the *g(τ,ν,λ)* function against the composite survival index (*CSI*), the percentage of survival (*SUR%*) and the median survival time (*MST*) for the inoculum 10^7^ CFU.

	Regression coefficients (95% CI ±)			R^2^	R^2^_adj_	MSE
	a	b	c			
*MST*	0.162 (−0.071, 0.395)	−501.294 (−1054.08, 51.497)	367.81(−195.07, 930.7)	0.37	−0.12	11.85
*SUR%*	0.354 (0.122, 0.586)	−1562.45 (−2472.47, −652.43)	968.64 (80.79, 1856.48)	0.82	0.68	162.4
*CSI*	−0.511 (−0.741, −0.281)	1887.502 (983.5, 2791.51)	−988.59 (−1782.6, −194.6)	0.91	0.84	0.007

MSE, mean squared error;

While regression analysis revealed a weak correlation between *MST* and the growth properties (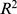 = 0.36; Fig. 3A), a strong correlation was found for the other two responses (for *SUR% R^2^* = 0.84, for *CSI R^2^* = 0.92; Fig. 3B & 3C).

Regression coefficients *a*, *b*, and *c* for the growth parameters *τ, ν* and *λ* (Equation3), respectively, associated with *CSI*, were as follows: *a* = −0.51 (95%CI: −0.72, −0.29), *b* = 1835.78 (95%CI: 1043.1, 2628.5), *c* = −526.92 (95%CI: −1009.6, −44.23). The negative coefficients *a* and *c* indicate a negative relationship between the respective growth characteristics *τ* and *λ* and the *CSI*. This means that the longer the lag phase and the higher the decay constant, the lower the *CSI* value. In other words, strains with a low *CSI* value are less virulent. The positive coefficient *b* indicates a positive relationship between the growth rate *ν* and *CSI*, which means that strains with a high growth rate have high *CSI* values.

The linear combination *g*(*τ,ν,λ*) of the growth parameters (Equation 3) combined with the estimated regression coefficients for *CSI* provides a good measure of the virulence of any given *A. fumigatus* isolate. Accordingly, an isolate with *g*(*τ,ν,λ*)* = *0, will have a *CSI* of 0.5, which corresponds to 50% *SUR%* and an *MST* of 15 days. This is also the case for any other combination of *SUR%* and *MST* that results in a *CSI* of 0.5 based on Equation 2. By definition, *CSI* is always less than or equal to 1-SUR%. Thus, a *CSI* of up to 0.5 is obtained only with a *SUR%* of at least 50%.

Figure 3B and 3C show, for the *SUR%* and *CSI* models, that the virulence of the two azole-resistant isogenic isolates, R1 and R2, was reduced relative to the other isogenic azole susceptible isolates and all other strains. Specifically, the *SUR%* in mice exposed to infection by R1 and R2 was greater than 60% and *g(τ,ν,λ)* was greater than 0. Two other isolates with mutations in *cyp51A* (TR_34_/L98H_45-07 and M220K) were less virulent than all WT isolates and the other *cyp51A* mutants. Eleven out of 15 isolates displayed a high *CSI* of 0.9, which corresponded with an increased probability of death when the value of *g*(*τ,ν,λ*) exceeded 2.2.

To verify whether all growth characteristics were significant for the *CSI* model, we additionally performed multiple regressions on models with reduced numbers of growth parameters. The F-test comparison of the original and nested models revealed that a reduction in each parameter resulted in a significant decrease in the performance of the model (Table 4). Table 4 also shows that extending the original model by *Y_0_* parameter did not lead to statistically significant improvement of the fit.

### The Novel Composite Survival Index is the Superior Virulence Marker

**Figure 4:**
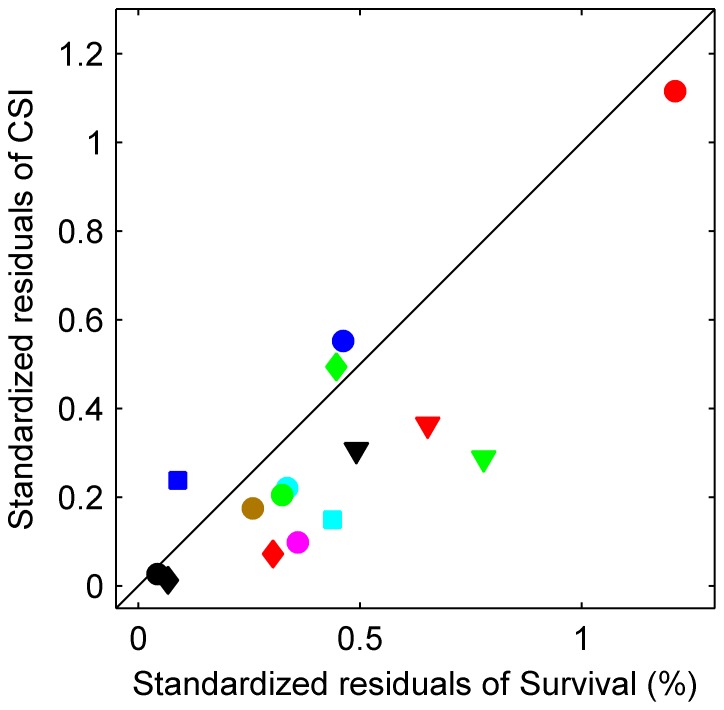
Leave one out-cross validation analysis. The *CSI* model describes the relationship between fungal growth and virulence better than the *SUR%* model because 12 of the 15 isolates had less standardized residuals for *CSI* than for *SUR%* (markers below the diagonal) (p<0.02). The shapes and colors of the symbols used for the observed data represent the same isolates as those defined in Figure 3.

We performed a comparative leave-one-out cross-validation study between the *SUR%* and the *CSI* models to verify the superiority of *CSI* over *SUR%*. Figure 4 demonstrates that the *CSI* model describes the relationship between fungal growth and virulence better than the *SUR%* model because 12 of the 15 isolates had less standardized residuals for *CSI* than for *SUR%* (Fig. 4, markers below the diagonal). To test the significance of the improvement in the residual values, we applied a binomial statistical test, which showed that the *CSI* model was significantly better than the *SUR%* model (*p*<0.02).

### Prediction of Virulence via *CSI*

**Figure 5:**
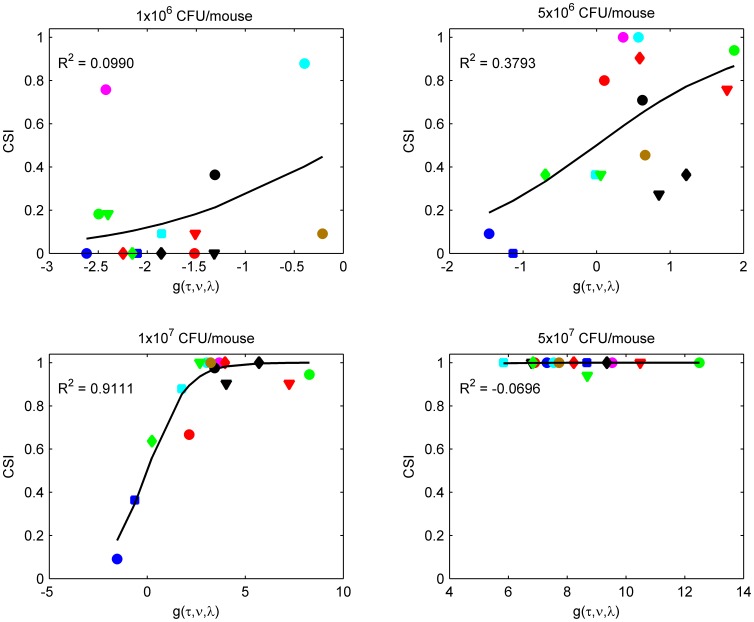
Relationship between *CSI* and *g*(*τ,ν,λ*) by systemic infection of mice with four different inocula. No clear sigmoidal relationship was found between *CSI* and *g*(*τ,ν,λ*) for the groups infected with 1×10^6^, 5×10^6^, and 5×10^7^ CFU per mouse. Changes in the virulence of each single strain due to different inocula indicate that *CSI* was also dependent on the degree of infection by the inoculums.

Mice survival for the aforementioned studies was assessed following an infection of 10^7^ CFU per mouse. To further confirm the validity of *CSI*, we carried out the infection in groups with three additional doses of conidia. Figure 5 depicts the resulting *CSI* versus the function *g*(*τ,ν,λ*) relationships for four different inocula. There was no clear sigmoidal relationship between *CSI* and *g*(*τ,ν,λ*) for the groups infected with 1×10^6^, 5×10^6^, and 5×10^7^ CFU per mouse. In panel with an infection of 10^6^ CFU, only the initial, shallow part of the sigmoidal shape of the *CSI* curve is apparent, whereas increasing the concentration to 5×10^6^ results in a steeper gradient of the curve. Lastly, the highest infection concentration of 5×10^7^ was associated with reaching the plateau of the sigmoidal curve and results in no differentiation between the different strains.

As expected, different inocula affected the virulence of individual isolates, indicating that *CSI* was also dependent on the inoculum size. Therefore, we defined a general model for predicting the virulence of a strain based on the initial inoculum (*Φ*) and the growth of the strains (Fig. S3; Equation 4).

Nonlinear multiple regression analysis showed the ability of the model to predict virulence, as shown by the good overall statistics of the fit (*R^2^* = 0.82, *p*<0.001; Fig. S3). The mean (95%CI) estimates of the regression coefficients a, b, c, d and the EC_50_ was −0.06 (−0.10, −0.01), 207.03 (111.45, 307.24), −156.42 (−266.68, −58.31), 3.81 (2.74, 5.96) and 6.60 (6.01, 7.13), respectively.

## Discussion

The primary aim of our study was to determine the impact of acquisition of azole resistance by *A. fumigatus* on virulence and to determine which *in vitro* growth characteristics are critical for *in vivo* survival. Our *in* *vivo* experiments showed that virulence, expressed as *SUR%* and *MST,* is variable, even between wild type isolates, and that azole-resistant clinical *A. fumigatus* isolates with *cyp51A* mutations are not less virulent. However, development of azole resistance may be associated with loss of virulence as was apparent with the set of four isogenic isolates, where the transition from an azole-susceptible phenotype to an azole-resistant phenotype was associated with higher *SUR%* and *MST*. A novel non-*cyp51A* mediated resistance mechanism was recently reported in these isolates, which consisted of a mutation in the CCAAT-binding transcription factor complex subunit HapE [40].Although the ergosterol biosynthesis pathway is critical for growth and proliferation of the fungus (and thus is an important drug target), it has previously been shown that SNPs in the *cyp*-gene of *Candida* occurred without major perturbation of the haem environment or activity, and as a consequence, allowed resistant mutants to produce ergosterol and retain fitness [41]. Our study suggests that this may also be the case in *A. fumigatus* that harbor SNPs in *cyp51A*. Although the exact role of HapE is not yet understood, isolates with the HapE mutation exhibited altered growth characteristics, such as impaired growth, suggesting that this mutation has implications for a broad range of processes in the fungal cell.

From a clinical perspective our observations are of importance as they indicate that *A. fumigatus* isolates with an azole-resistant phenotype due to a *cyp51A*-mediated resistance mechanism are capable of causing a similar spectrum of azole diseases as the wild-type isolates [10], [11], [12], [21], [28], [42].

This supports the clinical experience of increasing reports of cases of non-invasive and invasive aspergillosis due to azole-resistant isolates, and the high probability of failure during azole therapy [12], [43]. Furthermore, for TR_34_/L98H mutations, which are considered to have developed through exposure to 14α-demethylase inhibitors (DMIs), any fitness loss would be an important disadvantage when competing with wild-type field isolates. Our findings indeed indicate that the virulence of TR_34_/L98H isolates is comparable to wild type controls [8] as there is no growth impairment and thus no reduction of virulence.

In order to correlate *in vitro* growth characteristics with the *in vivo* markers of virulence a mathematical model was developed that indicated that three growth parameters, the lag phase, the growth rate (ν) and the decay constant (*λ*) were critical for virulence. Our model elucidates how these three parameters interact with each other and are associated with virulence expressed as the *CSI*. For instance, a long lag phase (τ) and high decay constant (*λ*) resulted in a low *CSI* and in low virulence; whereas high *CSI*s and virulence was characterized by rapid growth (*ν*) and short lag phases. However, the individual parameters can vary between isolates with similar *CSIs*. The wild type isolate V52–76 had the same *CSI* as the G54W mutant (*g* = 3.4; *CSI* = 0.96), although isolate V52–76 was characterized by a lower decay constant and shorter lag phase than the G54W mutant. The growth rate of G54W, however, was faster compared to isolate V52–76, which compensated for the other two growth parameters. Overall, a *CSI* of 0.9 corresponded with a high probability of death when the value of *g*(*τ,ν,λ*) exceeded 2.2. Analysis of residuals showed that the *CSI* correctly predicted virulence of each strain with a difference between estimated and observed *CSI* to be ≤0.1 for almost all isolates except for one group infected with TR_34_/L98H (isolate V45-07) for which a greater than 0.1 residual was found (Table 5). This may indicate that other factors than growth may play role in virulence of this strain related with host and/or fungus. For example, immunogenic molecules on the fungal cell wall surface that affect docking and pathogen recognition result in reduced virulence [44], whereas spore production, spore decay, spore settlement, spore germination, and mycelia growth rate are important fitness components for fungi [36]. Interestingly, a change in virulence was also observed and correctly predicted by the model for the M220K isolate. The *CSI* was approximately 0.5 times lower than that of the wild type isolates and the other *cyp51A* mutants, and around 0.5 times greater than the least virulent isolates R1 and R2, indicating reduced virulence. If as stated above *cyp51A*-mutations have little or no fitness costs, differences in virulence such as observed in the M220K are probably due to changes in other genes or pathways, similar to those that may occur in wild type isolates. As we used clinical isolates and the resistance mechanism as selection criterion, the genetic background of our isolates will vary significantly. The use of recombinants would be an interesting next approach to further explore the contribution of individual SNPs on the virulence of *A. fumigatus*.

**Table 5:** Rank of the fifteen clinical *A. fumigatus* isolates used for the evaluation of virulence in a murine model of disseminated aspergillosis based on the observed *CSI*s.

Isolates (__ID number_)		*g(τ,ν,λ)*	Predicted *CSI*	Observed *CSI*
WT__V28–29_	5.6853		0.9967	1
TR_34_/L98H__V61–76_	3.8431		0.9814	1
G54W__V59–73)_	3.4642		0.975	1
G138C__V59–72_	3.2132		0.9621	1
S2__V67–37_	3.0882		0.954	1
M220I__V28–77_	2.7105		0.9363	1
WT__V52–76_	3.4303		0.9689	0.9758
M220V__V13-09_	8.4007		0.9997	0.9455
TR_34_/L98H__V52-35_	7.3526		0.9993	0.9030
WT__AZN 8196_	4.0605		0.9825	0.9030
S1__V67-38_	1.7368		0.8536	0.8788
TR_34_/L98H __V45-07_	1.9441		0.8944	0.6667
M220K__V59-27_	0.2702		0.5594	0.6364
R2__V67-35_	−0.6141		0.347	0.3636
R1__V67-36_	−1.6555		0.1769	0.091

The *CSI* may be a useful tool to determine the impact of mutations on virulence. Moreover, the use of a general model (Equation 4) allows prediction of virulence *in vivo*, thereby reducing the number of animals required in such experiments. We have applied this tool in our laboratory to allow us to calculate the inoculum required for studies; this has led to substantial savings in time and animals.

In our study, we employed a relative simple and conservative murine model of aspergillosis. We used a nonneutropenic model, although patients with invasive aspergillosis often suffer from neutropenia and the opportunistic fungus is believed to be only infective when patients carry immunodeficiencies in one way or another. Once the fungal growth overcomes the immune defense the progress of the disease starts. This bears similarity to the model used. Mice are susceptible to conidia in high amounts and by increasing the infection-dose mortality increased. We could therefore avoid the use of immunosupressors that has been described to alter *A. fumigatus* growth and thereby affect the interpretation of our results [25], [45]. Moreover, the long-term effects of immunosuppressors on the fungus or host are almost completely unknown. Regarding the route of infection, there are two reasons for having chosen the disseminated model as opposed to an inhalation model. First, this infection model is well established and allows excellent control of infection parameters (such as inoculum), whereas the pulmonary model comes in a variety of forms with several technical problems including less well controlled infection. Secondly, Thomas and Elkinton reported that virulence and infectivity can primarily be measured when experimental infections occur through the intravenous portal because it involves establishment and spread within the host [46].

Multiple factors require investigation in the context of comparative virulence. We here focused on one aspect, the growth characteristics and conclude that these have a major impact on survival and virulence in the process of disseminated aspergillosis. Specific virulent factors relevant to conidial entry into the lungs, binding or germination at that site were not tested in this model. However, previous reports demonstrated the complexity of virulence and suggested that this complex host-microbe interaction can only be measured by the use of a multivariate mathematical model which will comprise multiple potential microbial and host factors of this interaction [47]. To understand the differential response of the host to fungal challenge, it is necessary to expand upon this basis in future studies.

## Supporting Information

Figure S1 **Fitting of the** ***in vitro*** **growth curves of fifteen** ***A. fumigatus*** **isolates**. The proposed model (Equation 1) for simulating the growth of *A. fumigatus in vitro* (blue line) fitted well to the observed growth curves (red line) of fifteen clinical isolates with *R* ^2^ values ranging from 0.98 to 0.99.
(TIF)

Figure S2 **Distribution of the wild-type population and populations with diverse** ***cyp51A*** **mutations**. Azole susceptible isolates (wild-type population) are black colored; red color, TR_34_/L98H mutants; blue, isogenic resistant strains R1 and R2; cyan, isogenic susceptible strains S1 and S2; green, M220I, M220K, M220V, TR_46_/L98, G138C, G54W.
(TIF)

Figure S3 **Prediction of** ***CSI*** **based on the inoculum size and the growth characteristics**. Graphs depicting the goodness of fit (*R* ^2^ = 0.82, *p*<0.001) of the full general model predicting *CSI* based on the growth-curve parameters *τ*, *ν* and *λ* and the inoculum size *Φ* (the four inocula are depicted with different colors) for the fifteen *A. fumigatus* strains.
(TIF)

Table S1 **Characteristics of thirty clinical** ***A. fumigatus*** **isolates used in our studies.**
(DOC)

Table S2 ***In vitro*** **growth characteristics and growth phase fit of thirty clinical** ***A. fumigatus*** **isolates.**
(DOC)
