# Supplementary figures and images for: Composite Survival Index to Compare Virulence Changes in Azole-Resistant Aspergillus fumigatus Clinical Isolates
Source: PLoS One. 2013 Aug 26;8(8):e72280. doi: 10.1371/journal.pone.0072280 (PMC3753310; doi:10.1371/journal.pone.0072280)

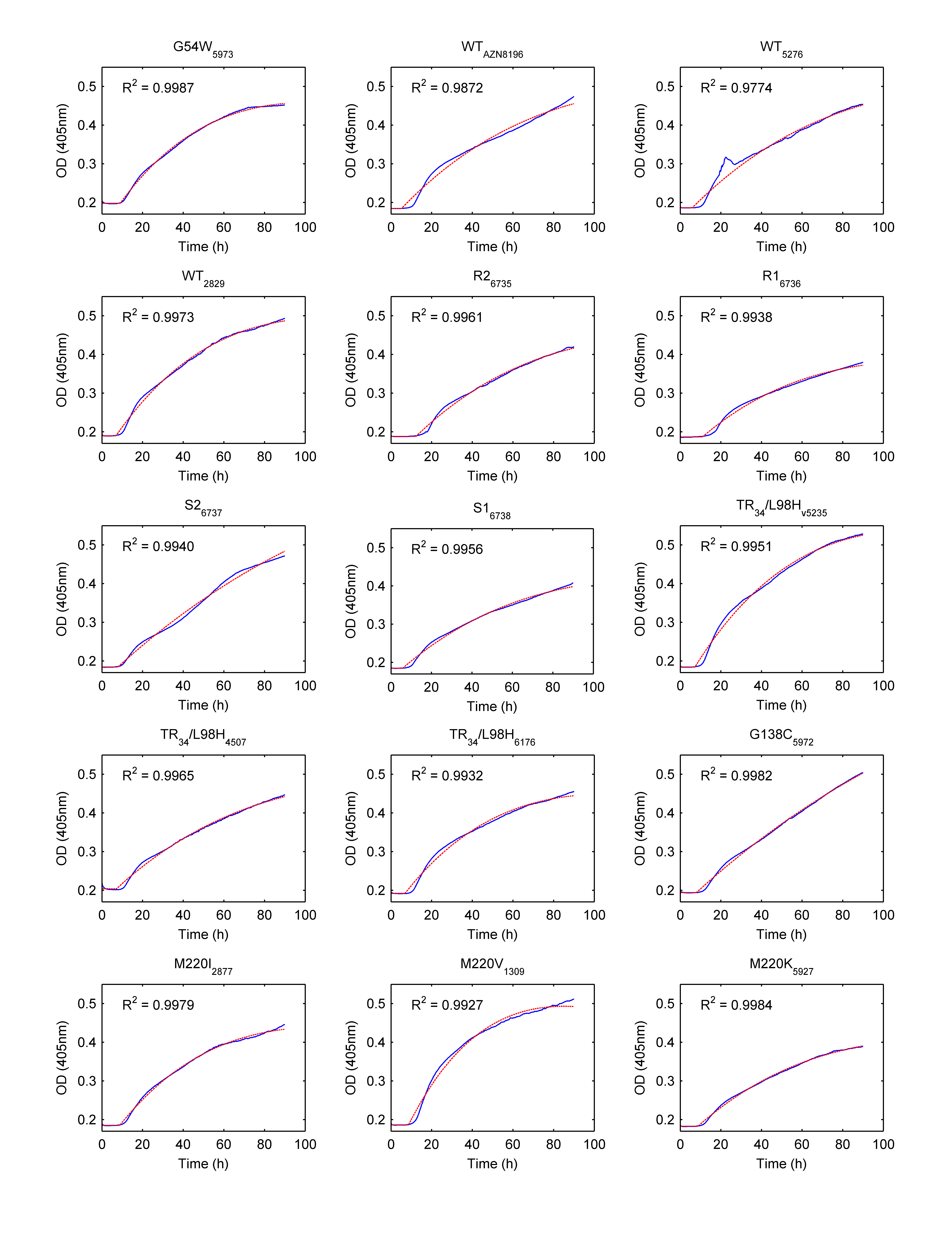

Supplement: Figure S1 — Fitting of the in vitro growth curves of fifteen A. fumigatus isolates. The proposed model (Equation 1) for simulating the growth of A. fumigatus in vitro (blue line) fitted well to the observed growth curves (red line) of fifteen clinical isolates with R 2 values ranging from 0.98 to 0.99. (TIF) [file pone.0072280.s001.tif]

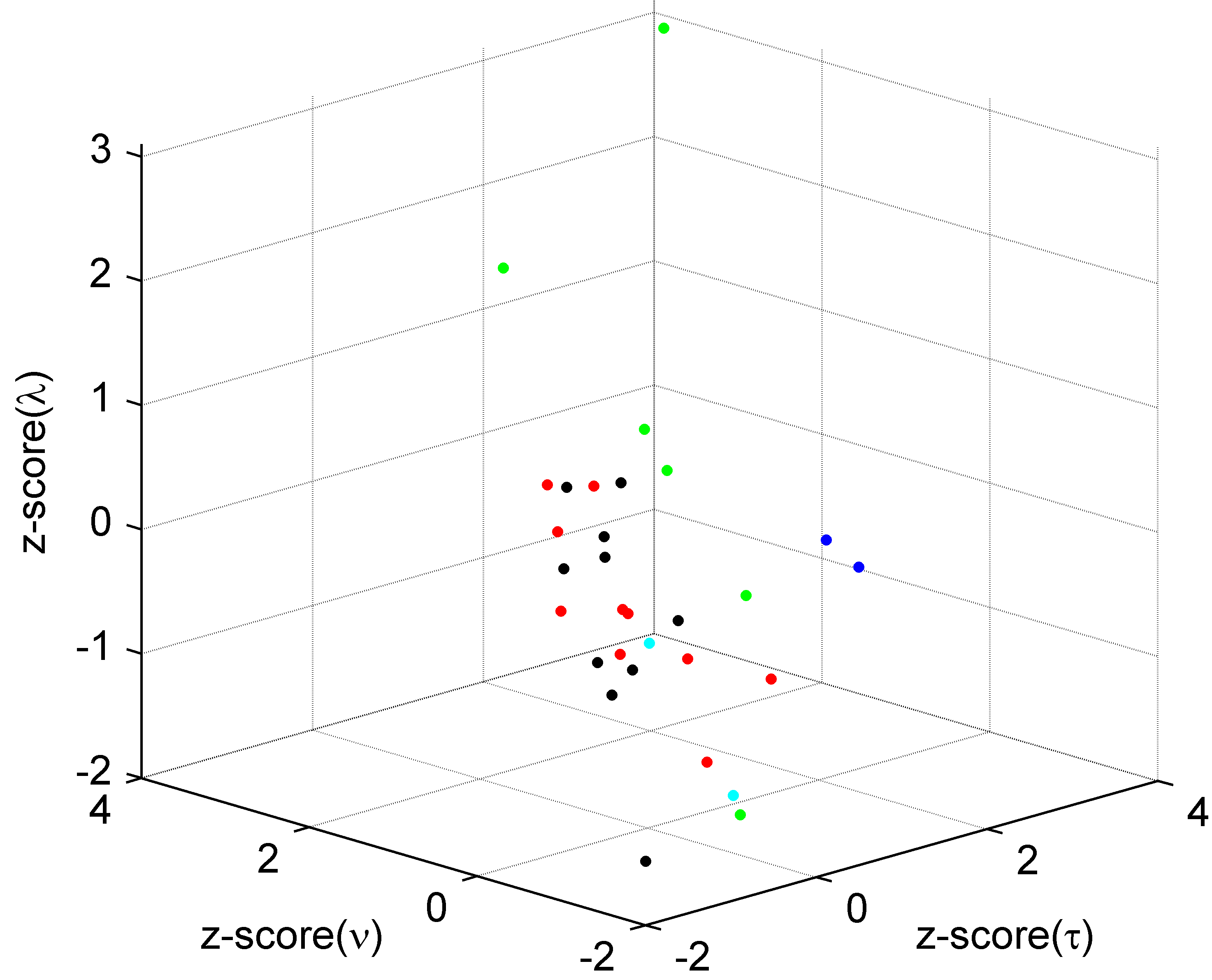

Supplement: Figure S2 — Distribution of the wild-type population and populations with diverse cyp51A mutations. Azole susceptible isolates (wild-type population) are black colored; red color, TR34/L98H mutants; blue, isogenic resistant strains R1 and R2; cyan, isogenic susceptible strains S1 and S2; green, M220I, M220K, M220V, TR46/L98, G138C, G54W. (TIF) [file pone.0072280.s002.tif]

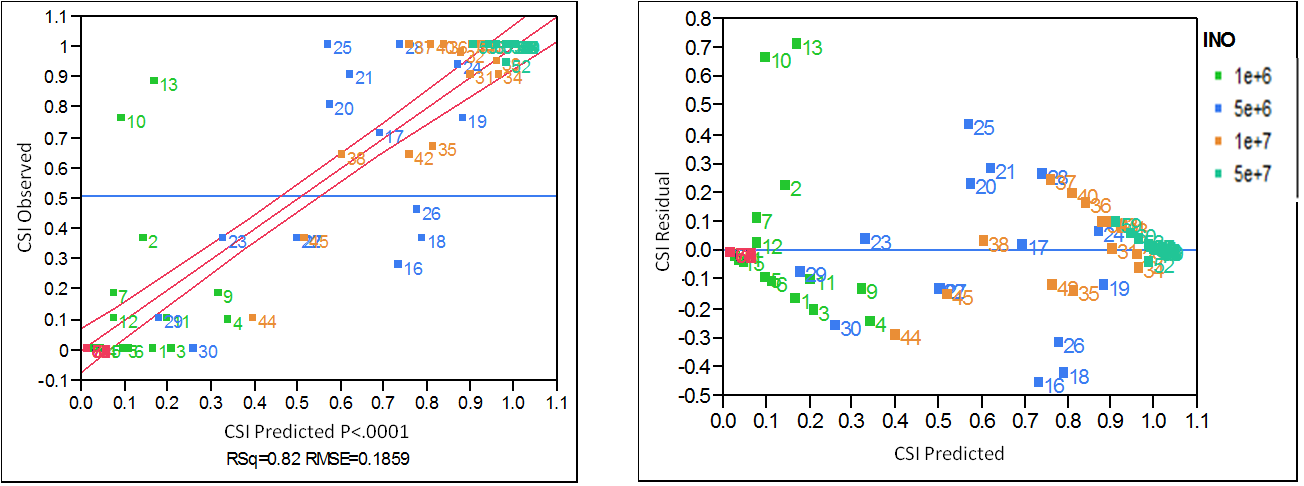

Supplement: Figure S3 — Prediction of CSI based on the inoculum size and the growth characteristics. Graphs depicting the goodness of fit (R 2 = 0.82, p<0.001) of the full general model predicting CSI based on the growth-curve parameters τ, ν and λ and the inoculum size Φ (the four inocula are depicted with different colors) for the fifteen A. fumigatus strains. (TIF) [file pone.0072280.s003.tif]
