# Supplementary material for: Composite Survival Index to Compare Virulence Changes in Azole-Resistant Aspergillus fumigatus Clinical Isolates
Source: PLoS One. 2013 Aug 26;8(8):e72280. doi: 10.1371/journal.pone.0072280 (PMC3753310; doi:10.1371/journal.pone.0072280)
Supplement: Table S1 — Characteristics of thirty clinical A. fumigatus isolates used in our studies. (DOC) [file pone.0072280.s004.doc]

**Table S1.** Characteristics of thirty clinical *A. fumigatus* isolates used in our studies.

| **Isolate ID** | **Origin^a^** | ***Cyp51A s*ubstitution** | **Short Tandem Repeats** | | | | | | **MIC (interpretation^b^)(mg/L)** | | |
| --- | --- | --- | --- | --- | --- | --- | --- | --- | --- | --- | --- |
|  |  |  | **3a** | **3b** | **3c** | **4a** | **4b** | **4c** | **ITZ** | **VCZ** | **POS** |
| AZN 8196 | Proven IA | (WT) None | 50 | 13 | 7 | 10 | 27 | 31 | 0.125 (S) | 0.25 (S) | 0.031 (S) |
| V54-73 | Possible IA | (WT) None | 29 | 17 | 7 | 10 | 26 | 5 | 0.25 (S) | 0.5 (S) | 0.063 (S) |
| V012-73 | Possible IA | (WT) None | 37 | 11 | 49 | 10 | 9 | 8 | 0.125 (S) | 0.5 (S) | 0.031 (S) |
| V030-17 | No Asp. Disease | (WT) None | 29 | 11 | 7 | 18 | 10 | 8 | 0.125 (S) | 0.5 (S) | 0.031 (S) |
| V033-63 | Proven IA | (WT) None | 49 | 11 | 7 | 13 | 9 | 5 | 0.063 (S) | 0.5 (S) | 0.031 (S) |
| V050-05 | Possible IA | (WT) None | 40 | 11 | 26 | 24 | 9 | 8 | 0.125 (S) | 0.5 (S) | 0.031 (S) |
| V52-07 | Proven IA | (WT) None | 38 | 11 | 46 | 10 | 9 | 8 | 0.125 (S) | 0.25 (S) | 0.031 (S) |
| V54-09 | Unknown | (WT) None | 35 | 11 | 7 | 25 | 26 | 5 | 0.125 (S) | 0.25 (S) | 0.031 (S) |
| V52-76 | Proven IA | (WT) None | 38 | 11 | 46 | 10 | 9 | 8 | 0.25 (S) | 1 (S) | 0.031 (S) |
| V28-29 | Proven IA | (WT) None | 25 | 23 | 21 | 14 | 8 | 5 | 0.125 (S) | 0.5 (S) | 0.031 (S) |
| V44-58 | No Asp disease | TR_34_/L98H | 36 | 9 | 9 | 8 | 10 | 11 | 16 (R) | 4 (R) | 1(I) |
| V64-51 | Proven IA | TR_34_/L98H | 32 | 9 | 6 | 8 | 10 | 20 | 16 (R) | 8 (R) | 1(I) |
| V99-47 | Proven CNS IA | TR_34_/L98H | 45 | 9 | 11 | 8 | 10 | 9 | 4 (R) | 16 (R) | 1(I) |
| V64-72 | Proven IPA | TR_34_/L98H | 32 | 9 | 6 | 8 | 10 | 20 | 16 (R) | 8 (R) | 1(I) |
| V77-40 | Chronic IA | TR_34_/L98H | 10 | 10 | 10 | 8 | 10 | 14 | 16 (R) | 16 (R) | 0.5 (I) |
| V79-79 | Unknown CE^c^ | TR_34_/L98H | 31 | 11 | 10 | 8 | 14 | 20 | 16 (R) | 16 (R) | 0.5 (I) |
| V80-01 | ABPA | TR_34_/L98H | 31 | 11 | 10 | 8 | 14 | 20 | 16 (R) | 16 (R) | 0.5 (I) |
| V52-35 | Proven IA | TR_34_/L98H | 84 | 11 | 7 | 9 | 10 | 11 | >16 (R) | 2 (I) | 0.5 (I) |
| V45-07 | Proven IA | TR_34_/L98H | 32 | 9 | 6 | 8 | 10 | 20 | >16 (R) | 8 (R) | 0.5 (I) |
| V61-76 | Proven IA | TR_34_/L98H | 85 | 9 | 10 | 8 | 11 | 21 | >16 (R) | 4 (R) | 0.5 (I) |
| V94-10 | Proven IA | 46 bp TR | 43 | 9 | 10 | 12 | 9 | 9 | 1 (S) | 16 (R) | 0.25 (S) |
| V28-77 | Aspergilloma | M220I | 37 | 29 | 33 | 10 | 9 | 8 | >16 (R) | 0.25 (S) | 0.5 (I) |
| V59-27 | Allergic PA | M220K | 15 | 9 | 18 | 17 | 9 | 5 | >16 (R) | 2 (I) | >16 (R) |
| V13-09 | Probable IA | M220V | 22 | 10 | 30 | 10 | 8 | 8 | >16 (R) | 1 (S) | 0.5 (I) |
| V59-73 | Unknown CE^c^ | G54W | 14 | 31 | 17 | 8 | 8 | 5 | >16 (R) | 0.125 (S) | >16 (R) |
| V59-72 | Aspergilloma | G138C | 36 | 9 | 9 | 2 | 10 | 26 | >16 (R) | 8 (R) | >16 (R) |
| V67-38 | chronic PA | (S1) None | 47 | 13 | 13 | 7 | 9 | 10 | 0.125 (S) | 1 (S) | 0031 (S) |
| V67-37 | chronic PA | (S2) None | 47 | 13 | 13 | 7 | 9 | 10 | 0.25 (S) | 2 (I) | 0.125 (S) |
| V67-36 | chronic PA | (R1) None | 47 | 13 | 13 | 7 | 9 | 10 | >16 (R) | 8 (R) | 0.5 (I) |
| V67-35 | chronic PA | (R2) None | 47 | 13 | 13 | 7 | 9 | 10 | >16 (R) | 8 (R) | 0.5 (I) |

**^a^**PA; pulmonary aspergillosis**,** IA; invasive aspergillosis, IPA; invasive pulmonary aspergillosis, ABPA; allergic bronchopulmonary aspergillosis, CNS; central nerve system; WT, Wild type (no *cyp51A* mutations and susceptible in azoles)

**^b^**Itraconazole and voriconazole: resistant (R) MIC > 2 mg/l, intermediate (I) = 2 mg/l, and susceptible (S) < 2 mg/l; posaconazole: resistant (R) > 0.5 mg/l, intermediate (I) = 0.5 mg/l, and susceptible (S) < 0.5 mg/l (28).

^c^CE; Clinical Entity
