# Supplementary material for: Composite Survival Index to Compare Virulence Changes in Azole-Resistant Aspergillus fumigatus Clinical Isolates
Source: PLoS One. 2013 Aug 26;8(8):e72280. doi: 10.1371/journal.pone.0072280 (PMC3753310; doi:10.1371/journal.pone.0072280)
Supplement: Table S2 — In vitro growth characteristics and growth phase fit of thirty clinical A. fumigatus isolates. (DOC) [file pone.0072280.s005.doc]

**Table S2**. *In vitro* growth characteristics and growth phase fit of thirty clinical *A. fumigatus* isolates

| **Isolate_ID number** | **Fitted Growth curve parameters based on the novel function^a^** | | | | | | | | | **Goodness of fit** | |
| --- | --- | --- | --- | --- | --- | --- | --- | --- | --- | --- | --- |
|  | ***τ ***(h) | | ***τ *(**95%CI **±)** | ***ν*** (OD x h^-1^) | | ***ν*** (95%CI **±**) | ***λ***(h^-1^) | | ***λ***(95%CI **±)** | **R^2^** | **R_adj_^2^** |
| WT_AZN 8196 | 5.1938 | 4.2147-6.1729 | | 0.0065 | 0.0063-0.0068 | | 0.0057 | 0.0053-0.0061 | | 0.9872 | 0.9871 |
| WT_V54-73 | 3.962 | 3.0883-4.8357 | | 0.004 | 0.0039-0.0041 | | 0.0029 | 0.0026-0.0031 | | 0.9947 | 0.9946 |
| WT_V012-73 | 6.6422 | 6.1838-7.1006 | | 0.0057 | 0.0056-0.0058 | | 0.0063 | 0.0061-0.0065 | | 0.9963 | 0.9963 |
| WT_V030-17 | 5.369 | 4.6714-6.0666 | | 0.0063 | 0.0061-0.0065 | | 0.0051 | 0.0048-0.0054 | | 0.994 | 0.9939 |
| WT_V033-63 | 6.5116 | 5.8808-7.1425 | | 0.0076 | 0.0074-0.0078 | | 0.0071 | 0.0068-0.0074 | | 0.9923 | 0.9923 |
| WT_V050-05 | 5.6616 | 4.8572-6.4661 | | 0.0079 | 0.0076-0.0082 | | 0.007 | 0.0067-0.0073 | | 0.9886 | 0.9885 |
| WT_V52-07 | 5.7511 | 4.9656-6.5366 | | 0.0069 | 0.0067-0.0071 | | 0.0077 | 0.0074-0.0081 | | 0.9869 | 0.9868 |
| WT_V54-09 | 6.5702 | 6.076-7.0644 | | 0.0072 | 0.007-0.0074 | | 0.0085 | 0.0082-0.0087 | | 0.9956 | 0.9955 |
| WT_V52-76 | 5.9517 | 5.2553-6.6481 | | 0.0063 | 0.0061-0.0065 | | 0.0055 | 0.0052-0.0057 | | 0.9774 | 0.9772 |
| WT_V28-29 | 6.9742 | 6.6278-7.3207 | | 0.0091 | 0.009-0.0092 | | 0.008 | 0.0078-0.0081 | | 0.9973 | 0.9973 |
| TR_34_/L98H_V44-58 | 5.4852 | 4.8077-6.1628 | | 0.0079 | 0.0077-0.0081 | | 0.0077 | 0.0074-0.008 | | 0.9909 | 0.9909 |
| TR_34_/L98H_V64-51 | 5.8983 | 5.1753-6.6214 | | 0.0065 | 0.0063-0.0067 | | 0.0065 | 0.0062-0.0068 | | 0.9913 | 0.9912 |
| TR_34_/L98H_V99-47 | 5.9626 | 5.3214-6.6039 | | 0.0064 | 0.0063-0.0066 | | 0.0064 | 0.0062-0.0067 | | 0.9931 | 0.9931 |
| TR_34_/L98H_V64-72 | 5.9696 | 5.4912-6.4481 | | 0.0043 | 0.0042-0.0043 | | 0.0042 | 0.004-0.0044 | | 0.9974 | 0.9974 |
| TR_34_/L98H_V77-40 | 8.1249 | 7.6583-8.5915 | | 0.0046 | 0.0045-0.0047 | | 0.0052 | 0.005-0.0054 | | 0.9966 | 0.9965 |
| TR_34_/L98H_V79-79 | 5.6905 | 4.9606-6.4204 | | 0.0064 | 0.0062-0.0066 | | 0.0058 | 0.0055-0.006 | | 0.9923 | 0.9923 |
| TR_34_/L98H_V80-01 | 5.0584 | 4.1878-5.9289 | | 0.0074 | 0.0071-0.0076 | | 0.0064 | 0.0061-0.0068 | | 0.9884 | 0.9883 |
| TR_34_/L98H_V52-35 | 7.261 | 6.7882-7.7338 | | 0.0099 | 0.0097-0.0101 | | 0.0078 | 0.0076-0.0081 | | 0.9951 | 0.9951 |
| TR_34_/L98 _V45-07 | 7.1825 | 6.7043-7.6607 | | 0.006 | 0.0058-0.0061 | | 0.0055 | 0.0053-0.0057 | | 0.9965 | 0.9964 |
| TR_34_/L98H_V61-76 | 6.8745 | 6.338-7.411 | | 0.0082 | 0.0081-0.0084 | | 0.0082 | 0.0079-0.0084 | | 0.9932 | 0.9931 |
| 46/TR_V94-10 | 15.952 | 15.8254-16.08 | | 0.0151 | 0.015-0.0152 | | 0.0134 | 0.0133-0.0135 | | 0.9991 | 0.9991 |
| M220I_V28-77 | 8.7829 | 8.4917-9.0741 | | 0.0081 | 0.008-0.0082 | | 0.0082 | 0.008-0.0083 | | 0.9979 | 0.9978 |
| M220K_V59-27 | 8.6958 | 8.4112-8.9805 | | 0.0058 | 0.0057-0.0059 | | 0.0064 | 0.0062-0.0065 | | 0.9984 | 0.9984 |
| M220V_V13-09 | 8.6418 | 8.2003-9.0832 | | 0.0125 | 0.0122-0.0127 | | 0.0109 | 0.0107-0.0112 | | 0.9927 | 0.9927 |
| G54W_V59-73 | 9.0146 | 8.7997-9.2295 | | 0.0089 | 0.0088-0.009 | | 0.0087 | 0.0086-0.0088 | | 0.9987 | 0.9987 |
| G138C_V59-72 | 8.002 | 7.5764-8.4273 | | 0.0053 | 0.0052-0.0054 | | 0.0027 | 0.0025-0.0029 | | 0.9982 | 0.9982 |
| S1_V67-38 | 5.8118 | 5.2844-6.3392 | | 0.0057 | 0.0056-0.0058 | | 0.0061 | 0.0059-0.0063 | | 0.9956 | 0.9956 |
| S2_V67-37 | 7.8409 | 7.0855-8.5963 | | 0.0053 | 0.0051-0.0055 | | 0.0031 | 0.0027-0.0034 | | 0.994 | 0.9939 |
| R1_V67-36 | 10.962 | 10.4441-11.4807 | | 0.0058 | 0.0056-0.006 | | 0.007 | 0.0067-0.0072 | | 0.9938 | 0.9938 |
| R2_V67-35 | 12.326 | 11.8861-12.7654 | | 0.0062 | 0.0061-0.0064 | | 0.0062 | 0.0059-0.0064 | | 0.9961 | 0.9961 |

^a^*** τ***, lag phase duration; ***ν***, growth rate; ***λ*** growth decay constant; OD, optical density.
